# Low Seroprevalence of Aichi Virus Infection in Taiwan

**DOI:** 10.3390/pathogens10050553

**Published:** 2021-05-03

**Authors:** Bao-Chen Chen, Tsi-Shu Huang, Nuan-Ya Huang, Chiao-Shan Chen, Yao-Shen Chen, Tsung-Hsien Chang

**Affiliations:** 1 Division of Microbiology, Department of Pathology and Laboratory Medicine, Kaohsiung Veterans General Hospital, Kaohsiung 81362, Taiwan; metapneumonvirus83@gmail.com (B.-C.C.); tshuang@vghks.gov.tw (T.-S.H.); nyhuang@vghks.gov.tw (N.-Y.H.); ccschen@vghks.gov.tw (C.-S.C.); 2 Department of Internal Medicine, Kaohsiung Veterans General Hospital, Kaohsiung 81362, Taiwan; 3 Faculty of Medicine, National Yang Ming Chiao Tung University, Taipei 11221, Taiwan; 4 Department and Graduate Institute of Microbiology and Immunology, National Defense Medical Center, Taipei 11490, Taiwan

**Keywords:** Aichi virus, seroprevalence, Taiwan

## Abstract

Aichi virus (AiV) belongs to the genus *Kobuvirus* of the family Picornaviridae; it is a single-stranded positive-sense RNA virus without an envelope. AiV causes acute gastroenteritis, abdominal pain, nausea, vomiting, and fever. Low incidence and high seroprevalence of AiV infections have been reported in several regions of the world; however, little was known on the prevalence of AiV infections in Taiwan. This study described the first two cases of AiV infection and analyzed AiV seroprevalence in Taiwan. A total of 700 sera were collected from a single hospital in southern Taiwan. The neutralization assay was employed to assess AiV neutralization antibodies in the serum. The test identified 48 positive cases, with a seroprevalence of 6.86%. Results also showed a gradual increase in AiV seroprevalence rate with age. Compared with other countries, Taiwan had a relatively low AiV seroprevalence, suggesting a low incidence of or sporadic AiV infections.

## 1. Introduction

Human Aichi virus (AiV) is a single-stranded, positive-sense RNA virus, a member of the *Kobuvirus*, *Aichivirus A* genus in the Picornaviridae family [1]. The virion shows a nonenveloped icosahedral morphology [2]. The AiV genome consists of about 8200 nucleotides. Flanked by a 5’ untranslated region (5’ UTR) and a 3’ UTR, the single open reading frame (ORF) of AiV encodes a polyprotein with a nonstructural leader protein, three capsid structural proteins P1 (VP0, VP3, and VP1), and seven nonstructural proteins (2A, 2B, 2C, 3A, 3B, 3C, and 3D), which are required for AiV replication [3,4]. Sequences in the VP1 and 3CD junction region have been used for AiV identification and genotyping. Currently, six different *Kobuvirus* species (*Aichivirus A–F*) within the *Kobuvirus* genus have been classified according to the 2019 Release of the ICTV (https://talk.ictvonline.org/ictv-reports/ictv_online_report/positive-sense-rna-viruses/w/Picornaviridae, accessed on 24 April 2021), and three genotypes (named A to C, respectively) have been identified within the human Aichi virus [1,5,6]. 

AiV causes acute gastroenteritis, with symptoms including diarrhea, abdominal pain, nausea, vomiting, and fever. AiV gastroenteritis is considered a viral foodborne disease through the fecal–oral route; however, subclinical infections of AiV pathogenesis are common [1,7,8]. These subclinical infections do not require medical attention, resulting in underestimation of the real impact of AiV on human health [9].

AiV shows an enterovirus-like cytopathic effect (CPE) in cell culture, and the appearance of cell shrinkage is almost the same as that of enterovirus in morphology. There is no identification reagent available for AiV in clinical practice. Instead, the enterovirus immunofluorescence antibody kit is used for identification in general clinical laboratories; hence, AiV is often judged as an NPEV (nonpolio enterovirus) and regarded as an unclassified enteric virus, causing the isolation rate to be underestimated or neglected. 

Different countries reported varied incidences of AiV infection, such as 5% in Northwestern Spain, 0.5% in Finland, 2% in Sweden, 0.43% in Denmark, and 1.8% in China [10,11,12,13,14]; and the diagnosis of AiV mono-infection and co-infections was also described. In a Japanese survey, RT-PCR analysis showed Aichi virus RNA detected in 55% (54/99) of patients’ fecal specimens among 32.4% (12/37) of outbreaks of gastroenteritis [15]. In comparison with the diverse range of incidences of AiV infection, a high seroprevalence was often reported. Seroepidemiologic studies in Japan, Germany, France, Spain, and Tunisia showed an increasing seroconversion rate of AiV during childhood or adolescence (0.3–68%) and high seroprevalence (80–100%) in the population aged over 40 years [16,17,18,19,20]. These data suggest that AiV is a causative agent of pediatric diarrhea; the high seroprevalence in adults indicates a general circulation of AiV exposure in different human populations, which implicates an important role of AiV infection in gastroenteritis.

In Taiwan, the first AiV cases were diagnosed in 2010. After that, AiV has not been isolated. Investigation data on gastroenteritis cluster infections obtained by the Taiwan Central Disease Control Agency in the past ten years contained no AiV cases. The isolation rate of this virus in Taiwan seems to indicate that it is sporadic. However, AiV did appear in Taiwan. Therefore, to understand whether AiV has been prevalent in Taiwan in the past, a survey on serum antibodies was conducted to analyze the distribution of antibody titers in different age groups and the AiV infection status in Taiwan.

## 2. Results

### 2.1. AiV Isolation in Taiwan

In Taiwan, the first two AiV were isolated from two newborns aged 3 and 5 days in 2010 at Kaohsiung Veterans General Hospital. The patients were referred by the postpartum care confinement center and were initially diagnosed with acute pharyngitis, acute gastroenteritis, and herpangina. Their clinical symptoms included vomit, vesicle over the hard palate, and irritable crying. The younger patient excreted sticky stool. The laboratory data of the 3-day-old newborn showed fecal occult blood, coxsackievirus (Cox) B1-B6 IgG immunofluorescence test: ≥1000, negative blood and urine bacterial cultures, negative throat virus culture, and positive rectal virus culture; while those of the older patient showed Cox B1, B6 test: ≥1000, Cox B3-B5 IgG immunofluorescence test: 1:320 and *E. coli*: >100,000 CFU/mL in urine culture. 

The virus culture analysis yielded a negative throat virus culture and a positive rectal virus culture in Vero cells showing enterovirus-like CPE. CPE was not observed in other cell types used in the laboratory, such as human embryonal lung fibroblast (MRC-5), human rhabdosarcoma (RD), human lung adenocarcinoma (A549), rhesus monkey kidney (LLC-MK2), and Madin–Darby canine kidney (MDCK) cells. In those Vero cells, enterovirus integrated antibody (PanEV) fluorescence staining was positive, while immunofluorescence staining of *Enterovirus* typing was negative. RT-PCR analysis of *Enterovirus* CODEHOP (COnsensus-DEgenerate Hybrid Oligonucleotide Primers) and *Parechovirus* were negative. Primary determination suggested no enteroviruses in the specimens, which were then subjected to Aichi virus RT-PCR analysis with the three primer sets: VP0 region, 708 bp; 2C to 3A-3B junction, 557 bp; and 3C to 3D, 519 bp, and they all showed positive results.

### 2.2. Phylogenetic Analysis

Three genotypes of AiV (types A to C) have been identified. Epidemiological studies revealed a certain geographical distribution of AiV genotypes, indicating circulation of genotypes A and B in different Asian and European countries [1].

**Figure 1:**
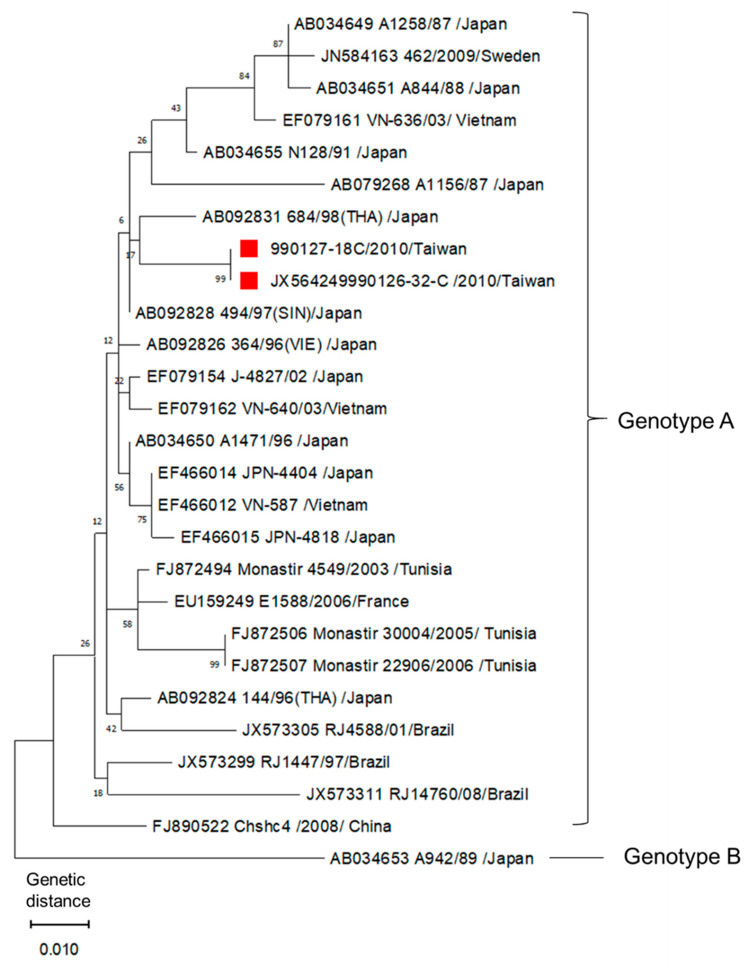
Phylogenetic analysis of AiV by comparison of 518 bp sequences in 3C/3D region using the maximum-likelihood method. The red marks indicate the two AiV isolates from Taiwan. Reference strains were obtained from GenBank, the accession number, strains, geographical origin of detection, and genotype are indicated. The tree with the highest log likelihood (−1569.54) is shown. The percentage of trees in which the associated taxa clustered together is shown next to the branches. Initial tree(s) for the heuristic search were obtained automatically by applying the maximum parsimony method. The tree is drawn to scale, with branch lengths measured in the number of substitutions per site.

Phylogenetic analysis on the two AiV isolates (990126-32 and 990127-18) of Taiwan was performed. Reference strains were obtained from GenBank. From the comparison with 518 bp sequences in the 3C/3D region, these two isolates were confirmed to be AiV type A. The two AiV isolates were 100% identical according to the sequencing results (Figure 1).

### 2.3. Seroprevalence Analysis

**Figure 2:**
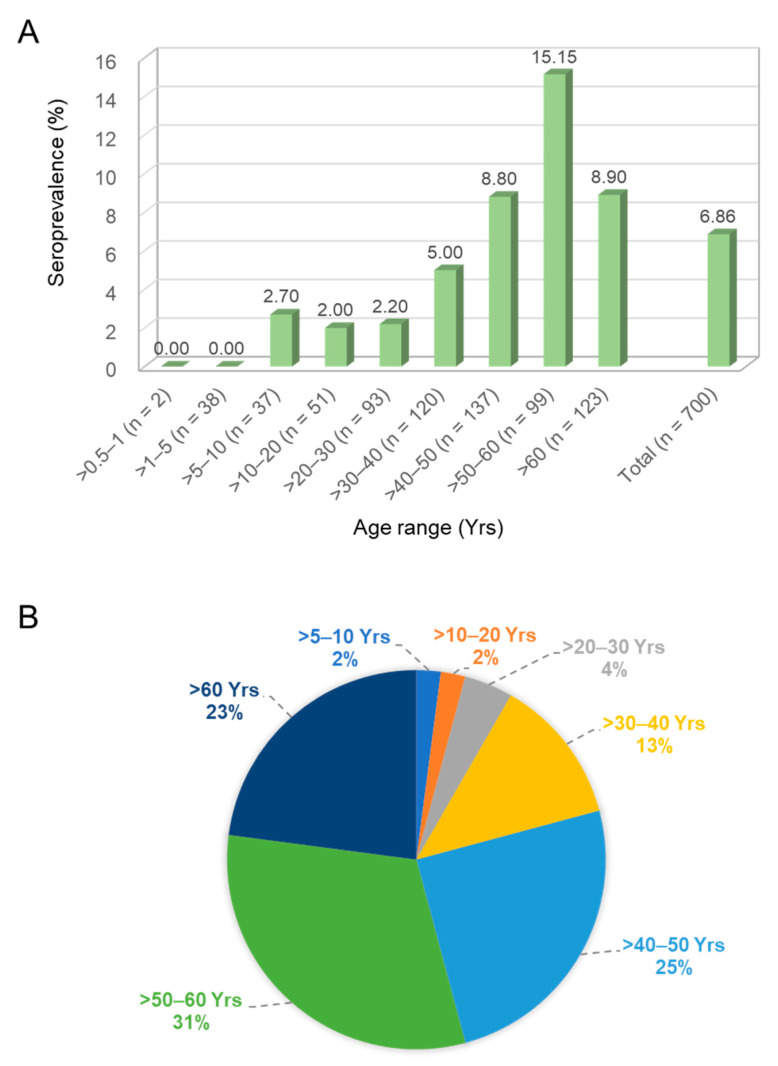
Seroprevalence of Aichi virus antibodies in a panel of 700 sera from Taiwan. (**A**) Number of cases (*n*) and percentage of positive samples in each age group in the total number of samples are given. (**B**) Age distribution of the 48 positive cases.

A total of 700 individual serum samples collected in a single hospital were enrolled and divided into nine age groups. The seroprevalence of AiV infection was surveyed using the neutralization assay against AiV-induced CPE. As previously defined [20], NT positive means an antibody titer exceeding 1:8 with neutralization activity. Of the 700 serum samples, 48 were NT positive, giving a total positive rate of 6.86%. The age group >50–60 years had the highest seroprevalence rate of 15.15% (15/99), and a reduction of seroprevalence was detected in the group aged over 60 (8.9%). In contrast, 0% seroprevalence was found in the age groups of >0.5–1 and >1–5 years (0/2 and 0/38, respectively) (Figure 2A). The trend of prevalence rate increased gradually from 0% to 15.15% in an age-dependent manner (chi-square test for trend = 14.76, df = 1; ***, *p* < 0.001). The correction test showed a similar result (Pearson’s correlation = 0.878; **, *p* = 0.002). The largest portion of the 48 positive cases was in the group of >50–60 years (31%) (Figure 2B).

## 3. Discussion

This study surveyed the seroprevalence of AiV in southern Taiwan using the neutralization assay. The overall prevalence was 6.86% and increased with age but showed differences in age distribution. The seroprevalence rate was 2.93% among those aged under 40 but rose to 10.58% among those aged over 40. These results show a low AiV prevalence in Taiwan compared to that of other regions.

According to the earliest report, the average positive AiV seroprevalence rate in Japan was 55.2% (460/833), and the positive rate increased with age, from 7.2% (9/125) among those aged >0.7–4 years to 87.5% (70/80) of those aged over 55 years. Almost 80% of all healthy adults acquired the neutralizing antibody by the age of 40 [20]. A survey conducted in Germany reported an AiV seroprevalence rate of 76% out of 485 samples [17]. It is worth noting that in Germany a high seroprevalence (51%) was detected in children under 2 years, and the rate then rose to >90% in the group aged over 40. A study in France showed that the seroprevalence rate was 25% in 0.7–9-year-old children and 85% in adults aged over 30 [16]. The average seroprevalence rate reported in the above studies was much higher than the present findings of Taiwan. Even Japan, another Asian country, showed eight times higher seroprevalence than Taiwan [20].

While Japan and Germany used the neutralization assay to survey AiV seroprevalence [17,20], the relationship between ELISA and neutralization assay was compared by France and Spain groups. Both methods, though different, yielded similar results and were comparable in surveying the antibodies against AiV in serum [16,18]. The serum neutralization assay can define the antibody activity against AiV, providing more information in the investigation of prevalence rate. Thus, the neutralization assay was adopted in our survey. However, the neutralization assay cannot detect non-neutralizing antibodies that may also be relevant for demonstrating previous Aichi virus infection and for titrating AiV-specific serum antibodies. Further investigation of seroprevalence using ELISA should be proposed in the future.

The first identified AiV cases in Taiwan were among infants; it would thus be of interest to understand the prevalence rate in young children. However, in this study, only 40 sera were collected from children under 5 years old, and none of them presented the AiV neutralization antibody. This prevalence rate was much lower than that obtained in Japan, Germany, and France [16,17,20]. Increasing the number of samples from those aged under 5 years would contribute to clarify the AiV infection status in Taiwan. 

Foodborne disease is a global health problem, AiV is a foodborne pathogen and is closely related to diet and environmental hygiene [1]. Foodborne events suspected to be linked to the consumption of raw seafood have been reported [21,22]. The low seroprevalence of AiV in Taiwan may be due to the prevailing diet [23]. Although Taiwan’s diet is internationally diverse, it is still mainly cooked food. AiV was reported to be inactivated by heat treatment at 56 °C for 20 min, resulting in a >4 log_10_ reduction on plaque assay [24]. A national estimation of disease burden from foodborne illnesses in Taiwan revealed that 53% of foodborne illnesses were caused by identifiable causal microorganisms. In addition to bacterial infection, norovirus was the leading viral pathogen associated with foodborne illnesses, followed by hepatitis A virus, and only a small portion of the enteritis cases were due to other viruses [23]. AiV has never appeared in that report, thus supporting the present findings that the positive rate of antibodies in all age groups is far lower than the results of other countries that prefer raw seafood [21,22].

Ever since AiV was isolated from the rectal swabs of two infants, there has been no other AiV isolation reported in Taiwan. Although there is no large-scale sudden infection in Taiwan, AiV can still appear sporadically. From the perspective of epidemic prevention, we cannot abandon AiV identification.

## 4. Materials and Methods

### 4.1. Virus and Cell Line

AiV strain kvgh99012632/2010 (accession no. JX564249) [3] was propagated in Vero cells (ATCC: CCL-81), which were cultured in Dulbecco’s modified Eagle medium (DMEM) supplemented with 10% FBS (Thermo Fisher Scientific, Waltham, MA, USA) at 37 °C and 5% CO_2_. The amplified virus was tittered by the assay of fifty-percent tissue culture infective dose (TCID50)/mL [24], and the virus was stored at −80 °C. Human embryonal lung fibroblast (MRC-5, ATCC: CCL-171), human rhabdosarcoma cells (RD, ATCC: CCL-136), human lung adenocarcinoma cells (A549, ATCC: CCL-185), rhesus monkey kidney epithelial cells (LLC-MK2, ATCC: CCL-7), and Madin–Darby canine kidney cells (MDCK, ATCC: CCL-34) were cultured in DMEM supplemented with 10% FBS at 37 °C and 5% CO_2_.

### 4.2. Laboratory Diagnosis

Virus identification involved the protocol for *Enterovirus* diagnosis of the Virology Group, Department of Microbiology, Kaohsiung Veterans General Hospital [25]. In brief, all swab specimens were collected using a transport medium containing Eagle’s minimum essential medium (EMEM) supplemented with antibiotics and 0.5% gelatin. Membrane filtration was performed to prepare rectal swabs. Rectal specimens were inoculated with the laboratory cell types, namely Vero cells, MRC-5 cells, RD cells, A549 cells, LLC-MK2 cells, and MDCK cells. Following the observation of CPE, samples with CPE were harvested and then fixed on the slides with acetone. *Enterovirus* group-specific immunofluorescence assay was performed with pan-*Enterovirus* antibody (Pan-EV, L66J, Thermo Fisher Scientific) according to the manufacturer’s protocol. 

The RT-seminested PCR (RT-snPCR) analysis method and primer design of all enterovirus serotypes were adapted from a previous report [26].

Clinical specimens were tested for *Parechovirus* RNA by RT-PCR according to our previous study [25]. PCR performed by amplifying a 760 bp fragment of the VP1 region using the following primer set: forward primer (5′-CCAAAATTCRTGGGGTTC-3′) and reverse primer (5′-AAACCYCTRTCTAAATAWGC-3′) [27]. The PCR products were analyzed by electrophoresis on a 1.5% agarose gel.

The procedure of RT-PCR analysis of Aichi virus in specimens was adapted from the previous report [15]. The PCR products (VP0, 2C to 3A–3B junction and 3C to 3D junction regions) were analyzed by electrophoresis on a 1.5% agarose gel.

Serum IgG antibodies against coxsackievirus type A (A7, A9, A16, and A24) and type B (B1, B2, B3, B4, B5, and B6) were detected by indirect immunofluorescence assay on biochips (EUROIMMUN Medizinische Labordiagnostika AG, Lübeck, Germany). Specimens were subjected to bacteria culture for identification using the Vitek 2 system (BIO MERIEUX, Marcy l’Etoile, France).

### 4.3. Phylogenetic Analysis

The PCR products of AiV 3C/3D region were purified using the GFX™ PCR DNA and Gel Band Purification Kit (Amersham Biosciences, Buckinghamshire, UK) according to the manufacturer’s instructions. The purified DNA fragments were sequenced and analyzed. Phylogenetic analysis of AiV was conducted according to the resulting sequences’ alignment in 3C/3D region using the maximum-likelihood method (Kimura 2-parameter model) with MEGA X software (https://www.megasoftware.net. accessed on 24 April 2021). There was a total of 518 positions in the final dataset. Codon positions included were 1st + 2nd + 3rd + noncoding. All positions with less than 60% site coverage were eliminated, i.e., fewer than 40% alignment gaps, missing data, and ambiguous bases were allowed at any position (partial deletion option). Because AiV is a globally distributed virus, 27 AiV isolates from different regions were chosen for the evolutionary analysis. The sequences of these reference strains shown in Figure 1 were obtained from GenBank. Except the two Taiwanese AiV strains, 24 genotype A and 1 genotype B isolates from different regions and year were used, which included early and later isolated AiV strains from Japan, Brazil, China, France, Sweden, Tunisia, and Vietnam. 

### 4.4. Neutralization Assay

The frozen preserved serum was placed in a 56 °C water bath for 30 min and then diluted with pH 7.0 PBS (1:4–1:512). Fifty microliters of diluted serum was mixed with 50 μL of virus solution (100 TCID50), and then placed in a 96-well flat-bottomed culture plate. The plate was incubated at 35 °C, 5% CO_2_ incubator for 3 h. Then, 100 μL of 2 × 10^5^ Vero cells were added into each well of the plate and cultured in the incubator at 35 °C, 5% CO_2_. The CPE was observed every day until 10 days. 

### 4.5. Statistical Analysis

Statistical analysis among the age groups were performed by chi-square for trend test and Pearson’s correlation test using the GraphPad Prism software (La Jolla, CA, USA). A *p* value less than 0.05 indicates statistical significance.

## Data Availability

The data presented in this study are contained within the article.
